# An Architecture and Management Platform for Blockchain-Based Personal Health Record Exchange: Development and Usability Study

**DOI:** 10.2196/16748

**Published:** 2020-06-09

**Authors:** Hsiu-An Lee, Hsin-Hua Kung, Jai Ganesh Udayasankaran, Boonchai Kijsanayotin, Alvin B Marcelo, Louis R Chao, Chien-Yeh Hsu

**Affiliations:** 1 Department of Computer Science and Information Engineering Tamkang University New Taipei City Taiwan; 2 Taiwan e-Health Association Taipei Taiwan; 3 Asia eHealth Information Network Hong Kong Hong Kong; 4 Standards and Interoperability Lab Smart Healthcare Center of Excellence Taipei Taiwan; 5 Department of Information Management National Taipei University of Nursing and Health Sciences Taipei Taiwan; 6 Sri Sathya Sai Central Trust Prasanthi Nilayam Puttaparthi India; 7 Thai Health Information Standards Development Center Health System Research Institute Ministry of Public Health Bangkok Thailand; 8 University of the Philippines Manila Philippines; 9 Taipei Medical University Master Program in Global Health and Development Taipei Taiwan

**Keywords:** blockchain, personal health records, health information interoperability, precision health care, health information management

## Abstract

**Background:**

Personal health record (PHR) security, correctness, and protection are essential for health and medical services. Blockchain architecture can provide efficient data retrieval and security requirements. Exchangeable PHRs and the self-management of patient health can offer many benefits to traditional medical services by allowing people to manage their own health records for disease prevention, prediction, and control while reducing resource burdens on the health care infrastructure and improving population health and quality of life.

**Objective:**

This study aimed to build a blockchain-based architecture for an international health record exchange platform to ensure health record confidentiality, integrity, and availability for health management and used Health Level 7 Fast Healthcare Interoperability Resource international standards as the data format that could allow international, cross-institutional, and patient/doctor exchanges of PHRs.

**Methods:**

The PHR architecture in this study comprised 2 main components. The first component was the PHR management platform, on which users could upload PHRs, view their record content, authorize PHR exchanges with doctors or other medical health care providers, and check their block information. When a PHR was uploaded, the hash value of the PHR would be calculated by the SHA-256 algorithm and the PHR would be encrypted by the Rivest-Shamir-Adleman encryption mechanism before being transferred to a secure database. The second component was the blockchain exchange architecture, which was based on Ethereum to create a private chain. Proof of authority, which delivers transactions through a consensus mechanism based on identity, was used for consensus. The hash value was calculated based on the previous hash value, block content, and timestamp by a hash function.

**Results:**

The PHR blockchain architecture constructed in this study is an effective method for the management and utilization of PHRs. The platform has been deployed in Southeast Asian countries via the Asia eHealth Information Network (AeHIN) and has become the first PHR management platform for cross-region medical data exchange.

**Conclusions:**

Some systems have shown that blockchain technology has great potential for electronic health record applications. This study combined different types of data storage modes to effectively solve the problems of PHR data security, storage, and transmission and proposed a hybrid blockchain and data security approach to enable effective international PHR exchange. By partnering with the AeHIN and making use of the network’s regional reach and expert pool, the platform could be deployed and promoted successfully. In the future, the PHR platform could be utilized for the purpose of precision and individual medicine in a cross-country manner because of the platform’s provision of a secure and efficient PHR sharing and management architecture, making it a reasonable base for future data collection sources and the data analytics needed for precision medicine.

## Introduction

### Background

Traditionally, standard clinics have offered medical services focused on disease treatment. However, with the world’s current aging populations, there is a growing gap between what services clinics offer and patients’ actual needs. This means that clinics may not be equipped to offer the complete range of care required by patients, resulting in preventable medical harm. The National Institute for Health and Care Excellence’s 2016 Multimorbidity Clinical Assessment and Management Guidelines Report [1] emphasized the importance of integrating patient-centered decision-making methods for multiple problems, with a focus on precision medicine. Precision medicine is a disease treatment and prevention strategy formulated with reference to individual variability in terms of genes, environment, and lifestyle, which is used to determine necessary dynamic changes and personalized treatment for preventative health care and clinical care. The core elements of precision medicine are historical disease data, daily vital signs data, personal health management, and medical record exchange, and it aims to stop potentially harmful or unnecessary medical behavior, integrate care, reduce treatment burden, and help patients select meaningful treatment and care goals through accurate assessment. With the requirements of precision medicine mentioned earlier, there is a need to not only maintain patients’ electronic medical records (EMRs) in hospitals but also to establish personal health records (PHRs) by combining medical records from different health institutes and functions of precision medicine, which patients can use to save, manage, use, and exchange with health care practitioners.

PHRs are highly private data, and this sensitivity means that there are significant security challenges involved in their management and exchange. Any system that seeks to manage and exchange such records must ensure that health records are exchanged appropriately, that they are not leaked, and that protected data are not tampered with. A good way to achieve the secure exchange of health records is by using blockchain architecture. A decentralized storage management architecture based on blockchain would be able to meet the security requirements. In a 2016 study, Ford [2] predicted that 75% of the adults worldwide will be using PHRs by 2020 without any external incentives. The importance of a PHR is that it allows a health care provider to examine a patient’s history of illnesses and medications and it provides a basis for medical decision making. More importantly, PHRs offer a basis for personal health management. PHRs include various health information such as medical information, vital signs (heartbeat, blood pressure, blood sugar, and body temperature), family disease history, and blood test reports [3-5]. Most countries today, however, still use the EMR system. In 2013 in Taiwan, a total of 502 hospitals had a comprehensive EMR system for accessing medical records, inspection reports, medical images, medication information, and so on. However, these data only exist in hospitals and are exchanged between other hospitals or clinics via the EMR exchange center. To achieve the goals of precision medicine and health care, a *patient-centered* approach to record management and exchanges is required; the traditional centralized PHR repository in hospitals does not meet the requirements to achieve this. A patient-centered approach would involve PHRs being managed by the patients themselves, while providing those records to various health care providers as needed. This kind of system would require a very secure architecture to protect PHR data.

According to the National Health Insurance (NHI) Administration of the Ministry of Health and Welfare in Taiwan, the average number of outpatient visits, not including Chinese medicine or dentists, is 13 per year for people in Taiwan. Most of these people visit different hospitals for treatment of the same condition over a short period of time. With the PHR system, people can manage their own health records and conditions, and doctors can also view their past medical records and medication status.

Blockchain technology was proposed by Nakamoto in 2008 [6] in a white paper titled “Bitcoin: A Peer-to-Peer Electronic Cash System.” A blockchain has the characteristics of decentralization, and its encryption mechanism can be designed to verify the data content to ensure that the data have not been tampered with. In this paper, the blockchain concept was used to solve the problem of data security and third-party authentication in the transaction process. A blockchain is a decentralized public account that records all money transactions and how much money everyone owns. John et al [7] proposed that the use of blockchain technology in electronic health care records can avoid the need to add another organization between the patient and the records. It is not a new repository for data but rather implies a decentralized control mechanism in which all users have an interest, but no one exclusively owns the data. This technology can improve data safety and remove privacy issues. Pouyan et al [8] stated that regarding the trust in health information exchange competency and exchange integrity, the blockchain architecture is more trustworthy than other exchange mechanisms for exchanging highly sensitive information.

This design differed from previous work on blockchain infrastructures and associated consensus mechanisms in that, while they operate in a decoupled manner from other blockchain frameworks, Fast Healthcare Interoperability Resource (FHIR) Chain [9] focuses on designing the decisions of smart contracts to be compatible with any existing blockchain architecture that supports the execution of smart contracts. However, this architecture remains vulnerable to the 51% of cyber attacks and does not provide complete data security.

### Objectives

This study proposed a blockchain-based architecture for storing, sharing, and protecting sensitive personal information. In the proposed architecture, the blockchain manages the authorization of data exchanges between patients, health care providers, and other users. The blockchain does not physically replace the electronic health record system, as most hospital information systems store detailed EMRs in a secure database on site or on a duplicate site located outside the hospital. Therefore, the blockchain architecture simply helps to ensure the security, confidentiality, integrity, and availability of the data. Combined with FHIR’s data format standards, stakeholders can read and write data into their own electronic health record systems that can be exchanged securely with other systems using the blockchain. The computational strength of the encryption built into the blockchain ensures that the data are correctly and safely transferred during PHR exchange transactions. However, a blockchain is not a data repository, rather it is a ledger of data integrity. This technology can be used to exchange records, verify data, and protect sensitive data. It can ensure that medical records will not be modified by unauthorized third parties. The uploading time of the data to the blockchain can also be recorded. Thus, the enabling of the collection of a patient’s more complete longitudinal data and the ability to share it remotely with professionals can allow for better decision making and reduce medical errors and medical malpractice.

## Methods

The blockchain-based exchange architecture for PHR management proposed in this study comprises 2 main components. The first component is the PHR management platform, on which users can upload PHRs, view their record content, authorize PHR exchange with doctors or other medical health care providers, and check their block information. When a PHR is uploaded, the hash value of the PHR is calculated by the SHA-256 algorithm, and the PHR is encrypted by the RSA (Rivest-Shamir-Adleman) encryption mechanism before being transferred to a secure database. The second component of the architecture is the blockchain exchange architecture, which is based on Ethereum to create a private chain. Proof of authority (PoA), which delivers transactions through a consensus mechanism based on identity, is used for consensus. The hash value is calculated based on the previous hash value, block content, and timestamp by a hash function.

**Figure 1:**
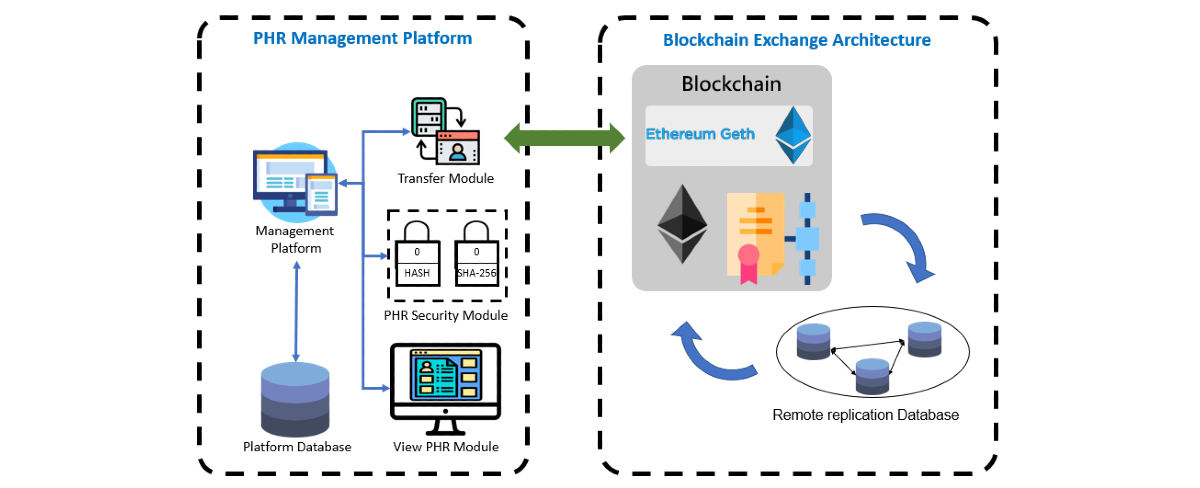
Personal health record management platform and blockchain architecture. PHR: personal health record.

The architecture of the platform is shown in Figure 1. The PHR management platform consists of the transfer module, the security module, and the view PHR module. The transfer module allows users to connect to the blockchain exchange architecture to create or search for blocks. The security module is used to encrypt and confirm the PHR content. The view PHR module displays the PHR content for personal health management or for doctors to view the record.

The blockchain architecture in this study is designed based on Ethereum, including elliptic curve digital signature, PoA, and the new block creation function. The blockchain architecture ensures that the PHR content remains secure and confirms that the PHR content is correct.

### Personal Health Record Management Platform

The major goal of this study was to build a cross-area health information exchange platform that could fulfill the needs of international medical services. This study used My Health Bank (MHB) as an initial example of PHRs. In Taiwan, MHB is issued by the NHI and contains a majority of the clinical data collected from different health care services. MHB not only includes the necessary clinical data chronically arranged by time for a single patient but also contains the information entered by the patient, such as blood pressure measured at home. Therefore, there was a good reason for this study to choose MHB for the PHRs in the Asia eHealth Information Network (AeHIN). Detailed items of the MHB are provided in this manuscript. Basically, PHRs refer to individual-centric personal health data from different medical service providers or devices, while EMRs represent the data of a patient in a single hospital.

Multiple simulated computers are used as blockchain nodes in this study to emulate the encryption and secure storage of a PHR in this study. As health records are private data, the blockchain must be built in a secure environment as a private chain, increasing the efficiency and stability of data transmission and sharing.

MHB was used as a PHR example in this study. MHB was launched by the Ministry of Health and Welfare of Taiwan in 2015. It allows Taiwan’s NHI members to download their own health records from its website.

The MHB data contain all the necessary clinical information because they are generated by the hospital when it applies for health insurance payments.

The entire PHR of any single patient was uploaded in our platform. For authority management and confidentiality, we used a variety of tags in the contents to specify the function levels to different uses through a carefully designed user interface, through which patients could assign which data would not be revealed to others as well as assign tags to the data. Our design to keep the whole data is for the purpose of future use of the data, as the PHR platform could also become a clinical data repository and the data could be used for further analysis of precision medicine in the future.

The MHB data include (1) outpatient information for Western medicine, traditional Chinese medicine, and dentistry; (2) hospitalization information; (3) allergy information; (4) images and information for pathological exams and tests; (5) patients’ discharge record abstract; (6) patients’ intention for organ donation and palliative care; (7) preventive health data; (8) preventive vaccination information; (9) patients’ health insurance card information; (10) premium and charging specific information; and (11) insurance premium payment specific information. The MHB file format can be selected as either XML or JSON. This study used the XML format.

#### Hash Value for Data Integrity Confirmation

To ensure that PHRs are not modified when they are transferred between platforms, this study designed a hash function to confirm the integrity of PHR data. SHA-256 was used to create a hash value for each PHR. SHA-256 is a cryptographic hash function, which takes an input and produces a 256-bit (32-byte) hash value known as a message digest, typically rendered as a hexadecimal number, 40-digit long. It was designed by the United States National Security Agency and is a US Federal Information Processing Standard [10,11]. If the PHR data have not been altered during transfer, the SHA-256 hash value would remain the same. Unlike encryption, which converts text into reversible cipher texts of different lengths, the hash function converts text into irreversible hash strings (or message digests) of the same length.

**Figure 2:**
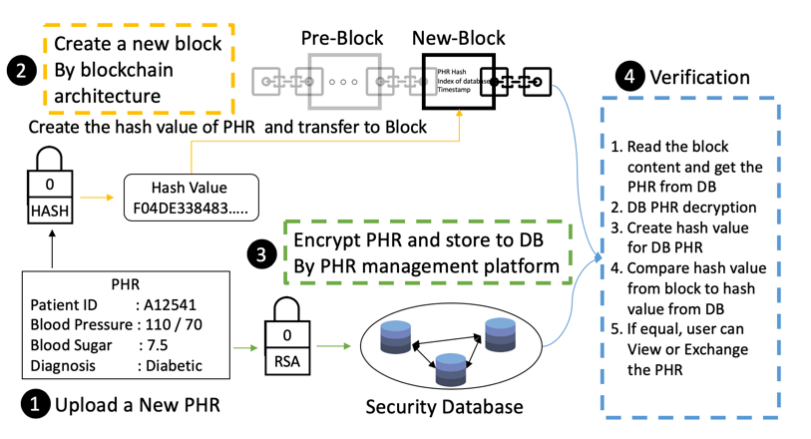
Personal health record creation, uploading, and verification procedure. DB: database; PHR: personal health record.

When users upload their PHRs to the platform, the PHR hash value is created and transferred to the blockchain architecture as block content. Then, when the PHRs are viewed by the owner, or exchanged with other users, the platform obtains the hash value from the block and calculates the PHR hash value by SHA-256 again. If the hash value from the PHR is equal to the hash value from the block, the PHR data have not been modified. The procedure of PHR management is shown in Figure 2.

#### Data Encryption for Personal Health Record Security

In this study, PHRs were encrypted by RSA before being uploaded to the secure database. RSA is a public-key cryptosystem used for secure data transmission [12]. The encryption key is public and differs from the decryption key, which is private. The platform automatically creates the RSA public and private keys for users. When users upload their PHRs, the public key is used to encrypt the record. Thus, even if a malicious attacker were to overcome the firewall and all other security mechanisms, they would only be able to obtain the encrypted PHR and would have no means of decrypting it. The user private key is used to decrypt the PHR when exchanged.

#### Viewing Personal Health Record and Block Information for Personal Health Management

This study designed a PHR exchange architecture in which PHR contents are not read when users upload their PHRs; the platform only uploads encrypted PHRs to the secure database, thus ensuring the security of personal data.

Moreover, this study developed a user interface for personal health management that shows PHR contents when users want to access them. Using MHB as an example, when users use the application to read their PHRs, it means that the platform has the authority to read the PHRs. The PHRs are then decrypted by the user’s RSA private key, and the platform reads PHR data, without storing them. This means that the platform cannot simply access PHRs without explicit user consent and action.

### Blockchain Exchange Architecture

**Figure 3:**
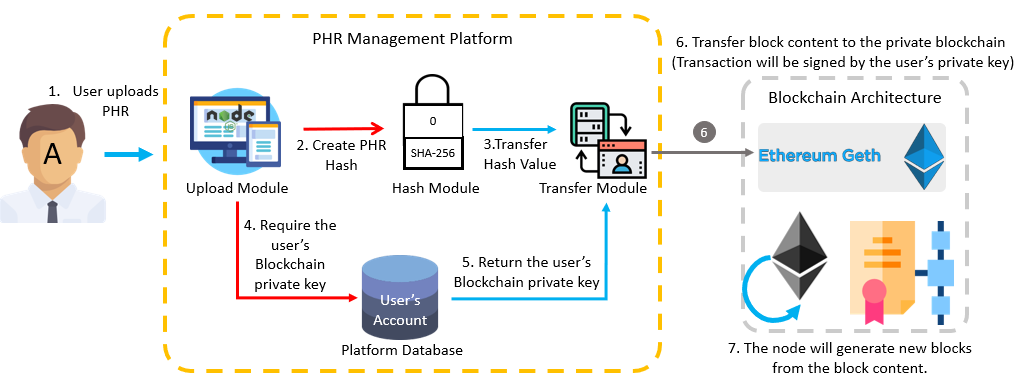
Block creation process. PHR: personal health record.

As the blocks in a blockchain cannot be tampered with or maliciously altered, this study stored PHR hash values in a blockchain to protect the PHR data and confirm the integrity of the PHR contents. Ethereum’s private chain was used as the blockchain architecture, and the Geth (Go Ethereum) application, which is the Ethereum protocol, was used to transfer the transaction from the proposed platform to the blockchain exchange architecture, create a new block, and connect to the blockchain. The block creation process is shown in Figure 3.

To secure against private data being leaked during transmission on the network, the data are encrypted during the data transmission process. The health record uploaded to the secure database by the platform is also encrypted to ensure the privacy of the user. The block content includes the PHR hash and timestamp, where the PHR hash is used to check whether the PHR in the database has been tampered with. If a malicious attacker attempts to obtain the block content, they will only get a collection of random numbers. The encryption method combines hash encryption and asymmetric encryption. The block content is protected by a hash encryption function that uses SHA-256 to scramble data into a set of hexadecimal strings. Asymmetric encryption uses the elliptic curve digital signature algorithm to encrypt PHR transfer information, ensuring the integrity and nonrepudiation of transaction data, and then the PoA consensus mechanism is used for validation by a qualified verifier established by an audited authority to confirm the correctness and validity of the PHR and create the verified blocks of the blockchain.

Elliptic curve cryptography (ECC) is a public-key cryptography based on elliptic curve mathematics, also known as asymmetric cryptography. The elliptic curve digital signature algorithm is based on ECC for digital signatures. The working principle is similar to that of most digital signature algorithms. They are signed with a private key and verified with a public key, thus offering nonrepudiation. Compared with traditional digital signature algorithms (such as RSA), ECC is faster, offers stronger security, and requires shorter signatures.

In the proposed platform, each user has one password for a user account and a private key for blockchain and PHR decryption. To improve the platform efficiency, users can choose to store their personal blockchain private key in the platform’s security database (or store it themselves). When data are uploaded to the platform, the system will retrieve the key from the database to complete the transaction process.

#### Proof of Authority for Block Creation

PoA is a technology that achieves consensus in a private chain. In the operation, an authorized node has the authority to generate the next block in a blockchain network. The blockchain information reaches the extreme value of the consensus of all nodes, which can guarantee that the latest blocks are accurately connected in series to the blockchain, and the blockchain information stored by the nodes is consistent, indivisible, and even resistant to malicious attacks. In this study, the private chain consensus mechanism is established, and the verifier is set on multiple simulated computers. The initial setup verifier node is set up on the simulated computers. In the feature, possible nodes represent cooperating institutions, medical institutions, research centers, and so on; the verifier uses this identity to obtain the right to verify.

Compared with other proof mechanisms, the key elements of the PoA network in this study include the following:

1. Improved efficiency: Block creation is accelerated and the waiting time for data exchange is reduced.
2. Verifier setup: A mutual supervision relationship with partner institutions is established to allow self-supervision and supervision of others, preventing the blockchain from being controlled by the node manager; the verifier can vote for a new verifier or remove an unqualified verifier at any time.
3. Highly scalable and highly compatible: It is also possible to complete intelligent collaborative construction and optimize it.

#### Hash Value for Block Corrected, Confirmed, and Connected

**Figure 4:**
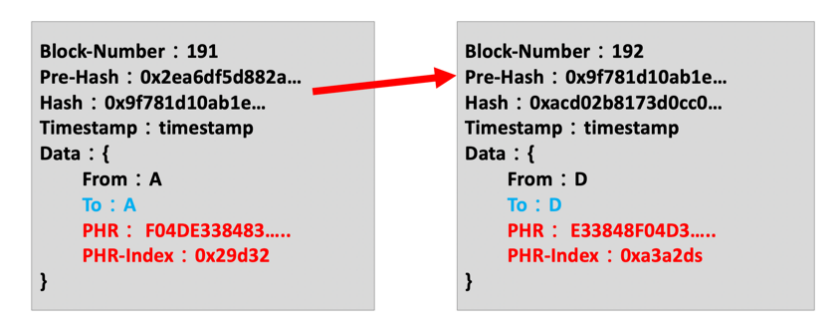
Block content on the blockchain architecture. PHR: personal health record.

The cryptographic hash function is an important part of the blockchain. It is essentially a function that gives security capabilities to the created block, based on processed transactions, making them immutable. In Ethereum’s function, SHA-256 is used to create new blocks. The hash of a block is created based on the block content, previous hash value, and timestamp. The block content and architecture are shown in Figure 4.

Block content includes the following:

- Block number: Current block number
- Pre-Hash: The hash value of the previous block
- Hash: The hash value of this block
- Timestamp: Current time
- PHR hash: The hash value of PHR created by the platform
- PHR index: The index position of the health record in the secure database

### Overall System Workflow

#### Personal Health Record Exchange Authority Mechanism

**Figure 5:**
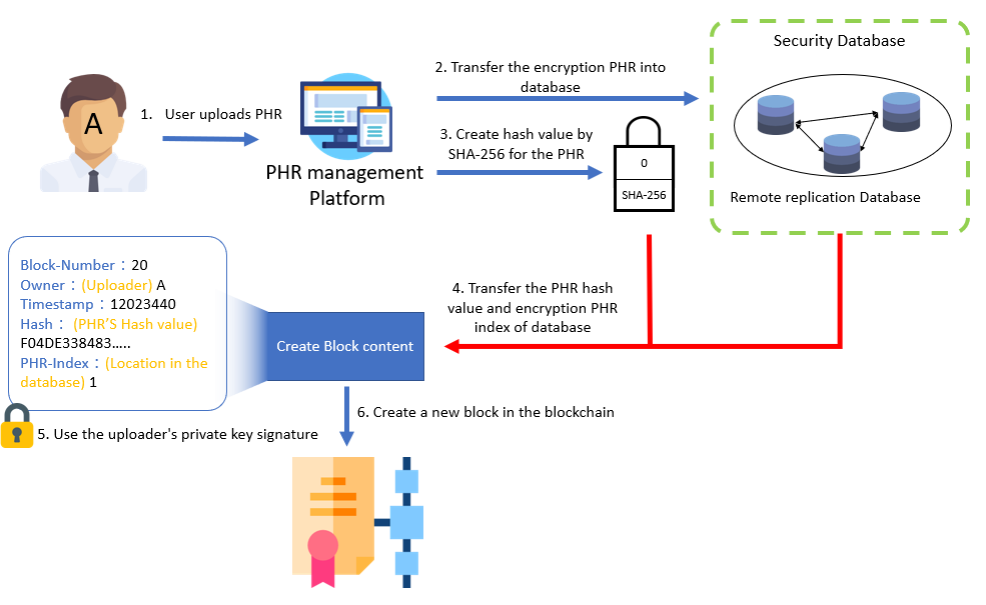
Workflow of a user uploading their personal health record. PHR: personal health record.

Users can manage the authority for PHR exchange once they have uploaded their PHRs. When users want to make their PHRs available to a doctor, the authority assignment procedure is as shown in Figure 5.

**Figure 6:**
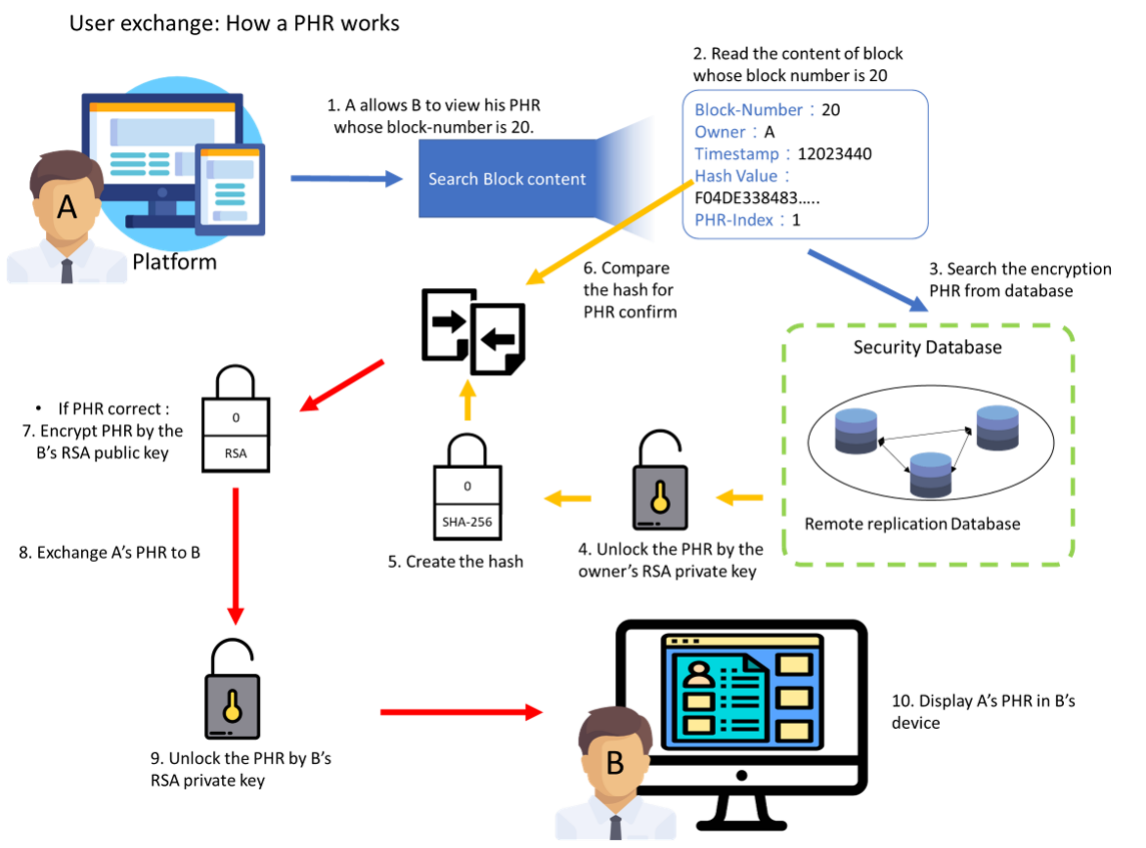
Workflow of a user sharing their personal health record with a doctor. PHR: personal health record; RSA: Rivest-Shamir-Adleman.

The workflow of the system comprises 3 components: upload, exchange, and view. To begin, a user uploads their PHR to the platform (Figure 6).

In the uploading process, the PHR is assigned a hash value by SHA-256. Then, the PHR is transferred to the secure database after encryption by RSA. Once the data are stored in the database, blockchain is used to ensure data security and integrity. SHA-256 and ECC are then used to create a block, and the Ethereum architecture is used as the blockchain architecture in this study. The PHR hash value and the PHR index in the database are transmitted to the Ethereum block by the user’s blockchain account (public key) and using the user’s private key signature. To create a block, block content must be verified and the block hash value must be calculated by the verifier node; it is then broadcast to each node.

The workflow of users sharing their PHRs with a doctor is shown in Figure 6. First, the platform sends the transaction to the blockchain architecture. The block architecture will then select the user’s block and read its content. The users’ PHRs will be obtained from the secure database based on the database index of the PHRs and decrypted using the users’ private key, and the hash value will be created again. The PHR will then be transferred to the doctor after being encrypted by the doctor’s RSA public key and decrypted by the user’s private key if the hash value is equal to the block content’s hash.

**Figure 7:**
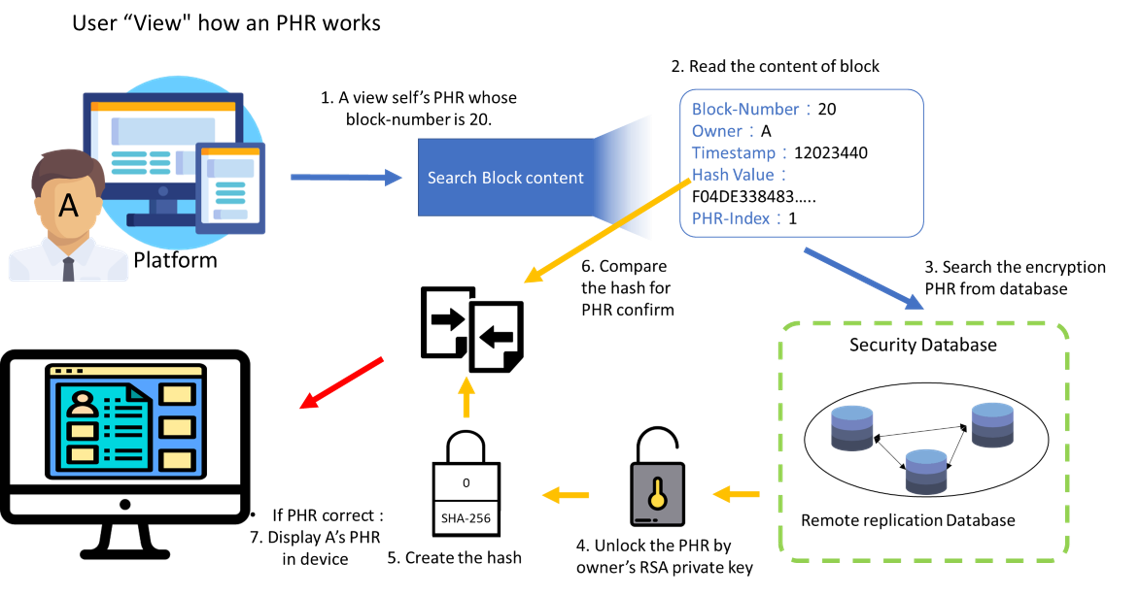
Workflow of a user viewing their own personal health record. PHR: personal health record; RSA: Rivest-Shamir-Adleman.

The workflow of viewing the PHR content is shown in Figure 7. When users want to view their PHRs or share their PHRs with a doctor, the platform will send the transaction to the blockchain architecture. The blockchain architecture will confirm that the PHR content has not been modified and that the user or doctor has the authority to view the PHR. The PHR will then be transferred to the user or doctor after being encrypted by their RSA public key. The user or doctor will then use their own private key to decrypt it. They will then be able to view the PHR content. MHB is used as an example in this study.

#### International Personal Health Record Exchange Implementation Process

The platform can be used at all places where the internet is available. This study used the data format designed in Taiwan MHB as an example to test the system in Asia. MHB contains all the necessary clinical health care data. In Taiwan, 99% of residents can access MHB. Therefore, we chose MHB as an example of PHRs for the AeHIN. The Philippines and Thailand were used as test cases for this study, and 2 of the physician representatives in this study were Dr Alvin in the Philippines and Dr Boonchai in Thailand.

A testing scenario was designed in which a patient from Taiwan travels to Bangkok and the Philippines and suddenly requires medical services. Both the patient and doctors in different countries were registered on this platform. Before the patient would see a doctor in a specific country, authorization to view the PHR would need to be given to the doctor by the patient. For this testing scenario, a patient’s PHR with diagnoses of type 2 diabetes mellitus, epilepsy, brain stem stroke, and proteinuria NOS (not otherwise specified) and medication data was designed. The data of testing scenario is descripted in Table 1.

The scenarios consisted of the following scenes:

1. A patient from Taiwan travels to the Philippines.

2. The patient develops a headache and dizziness.

3. The patient goes to see a doctor who has been registered in our platform.

4. Authorization to view the PHR is given to the doctor.

5. The doctor retrieves the patient’s PHR from the platform.

6. By viewing the previous PHRs of the patient, the doctor obtains the health profile of the patient and then completes a new diagnosis, treatment, or medication order according to the current status of the patient.

7. A new block is created and the new PHR is stored in the PHR database, if the doctor is willing to upload the new record.

**Table 1:** The data of testing scenario for international personal health record exchange.

Num	Date	Diagnosis	Medical
1	October 10, 2017	Type 2 diabetes mellitus	Iunaidon Tablets *Yu Sheng*
2	July 16, 2017	Epilepsy	Neurtrol F.C. Tablets 300 mg.
3	May 25, 2017	Brain stem stroke	Cofarin Tab 1 mg
4	May 20, 2017	Brain stem stroke	Cofarin Tab 1 mg
5	January 20, 2017	Proteinuria not otherwise specified	Kaluril Tablets 5 mg

## Results

### Study Design

**Figure 8:**
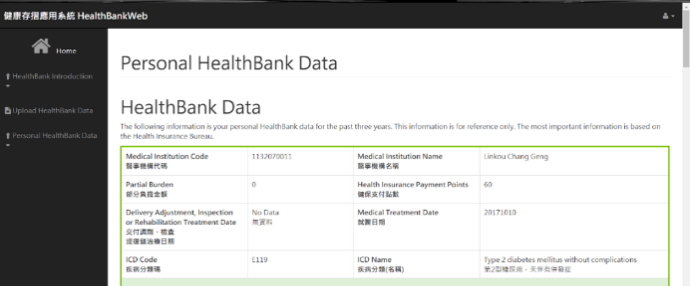
The user interface of a personal health record viewer.

This study designed a blockchain-based PHR exchange architecture and management platform for the secure management transfer and sharing of PHR data between patients and medical health care providers. In the PHR management component, the user interface was established; its functions include viewing PHRs for personal health management, sharing PHRs with a doctor, and checking the blockchain content for security. The PHR viewer user interface is shown in Figure 8. MHB was used as an example in this study.

### The Personal Health Record Viewer User Interface of Platform

In Figure 8, the uploaded PHR is displayed. Records are sorted in a time sequence from the latest to the oldest. The display shows the record of each visit, and the patient’s medication history. A doctor can view the latest related health record and recent medication status to give the patient the most appropriate diagnosis, while avoiding the problem of adverse reactions between repeated medications or adverse medications.

### Blockchain Information in the Platform

**Figure 9:**
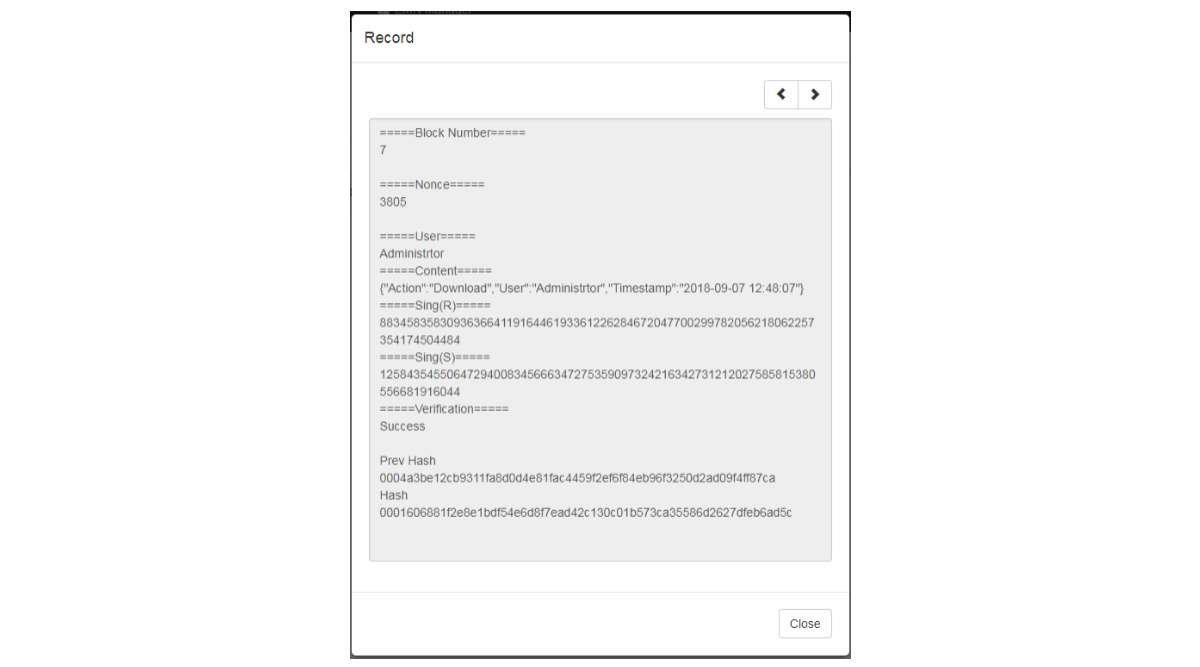
Block content.

The block content is shown in Figure 9 and includes the time at which the PHR was uploaded, the PHR owner, the PHR hash value, a timestamp, a block hash value, and a pre-hash value. Each block records the previous block location and concatenates to the previous block.

When users upload their personal MHB file, the system automatically converts the file to the FHIR format and transfers it to the security database. The data are then encrypted and uploaded to the blockchain. The users can view the uploaded data records and the contents of the block by uploading the module and obtain a health record for downloading in the FHIR format. A hospital can then upload that data to their system, as long as the system supports the FHIR format.

**Figure 10:**
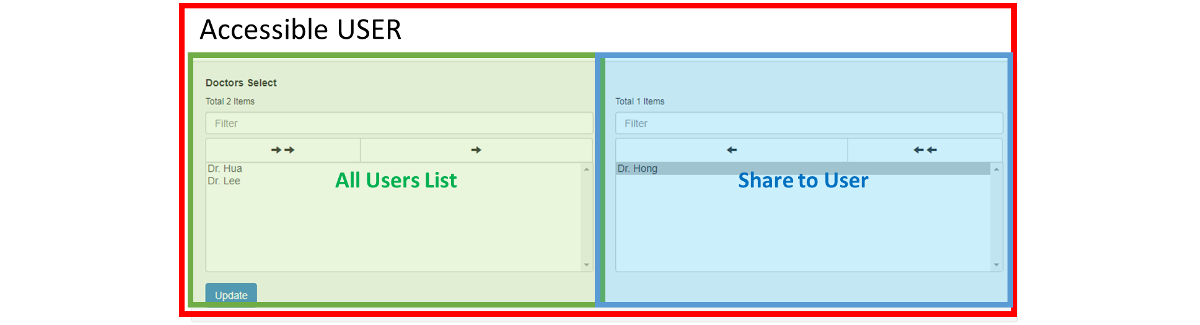
Authority control user interface.

The blockchain architecture allows users to set their own PHR read permission using the PHR management platform to control who can view their records. The blockchain is used to confirm that the PHR content is correct. The authority control user interface is shown in Figure 10. The simple user interface design ensures that the platform and function are easy to navigate and operate. The design uses 2 columns to display a list of permissions, one of which is a list of trusted participants, and the other is a list of participants to whom the user wishes to grant permission to view their current PHR. When the user wants to grant a doctor permission to view their PHR, they select the doctor from the left-hand column and update the identity.

**Figure 11:**
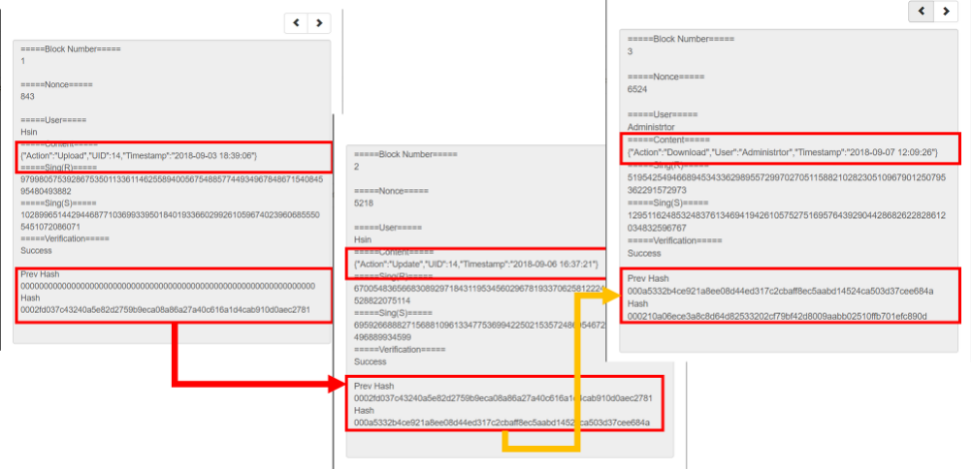
Blockchain connection diagram.

The blockchain architecture in this study is built by Ethereum, and the blocks are connected by the hash value of each block. The connection diagram is shown in Figure 11.

### Testing Feedback from Physicians

This study used the MHB data as an example to demonstrate the functioning of the PHR exchange platform and the PHR exchange mechanism based on the blockchain architecture and encryption mechanism, which can ensure PHR storage security and its tamper-proof nature.

The system can more effectively manage self-health records and provide physicians with PHRs as a decision-making reference. The results of this study have been cross-nationally tested in Southeast Asian countries, exchanging PHRs via the AeHIN, and invited physicians from Southeast Asian countries as international participant doctors to allow users to exchange PHRs internationally for appropriate treatment.

The proposed platform was designed to easily share and exchange PHR information electronically. The contents of the PHRs were protected and kept unchanged by the technology of the blockchain architecture. The international standard format of Health Level 7 FHIR was designed in this platform to ensure the interoperability. Doctors could use the platform to upload and download PHR data from different places at any time, thereby allowing PHRs to be exchanged efficiently. Therefore, this platform could increase the accessibility, interoperability, timeliness, and usability of PHRs.

The platform is currently in its testing stage, and there is a low number of users on the network. The users’ comments could be summarized as follows:

1. PHRs that are in a standardized format on this platform are a benefit for clinical service.
2. By using the platform, the exchange of PHRs is easy and efficient.
3. The protection offered by the blockchain technology can convince users that the system is secure.
4. Even if the role of the user is that of the platform manager, PHRs still cannot be read without the authorization given by the patient to view the PHR.
5. Personal health management functions can be designed in future work.

## Discussion

### Potential

Blockchain technology has great potential for electronic health records [13]. The core of the blockchain model ensures that any information involved has nonrepudiation to maintain the correctness of the historical process records [14]. Gary et al [15] Reviewed the current PHR definitions and multiple blockchain architectures for PHR management and found that blockchain technology is a key requirement for the management of consent to use private health data.

Many studies have proposed health applications based on the blockchain technology that can be used in the medical domain to achieve medical record sharing. In 2016, Ekblaw et al [16] created a decentralized medical record management platform that was built on the private network of Ethereum. The platform can only be accessed by authorized users, and blockchain was used to manage authentication, data sharing, and other security functions in the medical field. In the study, when any information was updated on the hospital side, it was uploaded to the blockchain; the platform was synchronized with the patient’s database, and the patient would be reminded to update the block. However, patients were unable to upload data themselves, as the data were all still stored in the centralized hospital database. Omar et al [17] used Ethereum’s smart contracts and a decentralized application to build a cloud-based PHR system. This system was used to store the PHR of each user and also to ensure the security and integrity of the uploaded data. Private accessible units (PAU) were responsible for all encryption, decryption, uploading of data, searching for data, and verification of data in which users can encrypt data with an encryption key and upload data to the blockchain through a smart contract, which then returns a block-id to the user uploading the data. The user would be responsible for remembering the block-id. To view the data, the user would provide the PAU with the block-id, and the system would automatically return the corresponding block content and decrypt it with the decryption key. This system, however, did not offer the capability of sharing personal medical records or system interoperability.

Peterson et al [18] presented a blockchain-based approach to sharing patient medical data that relies on a single centralized source of trust rather than network consensus to translate data and provides consensus on proof of structural and semantic interoperability. Zhang et al [9] presented a blockchain-based framework FHIR Chain that was designed to fit the technical requirements defined by the Office of the National Coordinator for Health Information Technology interoperability roadmap.

Precision medicine requires the accurate collection and management of all kinds of clinical data. To this end, this study constructed an innovative data storage mechanism, used blockchain technology to ensure the correctness and safety of the PHR data, and combined a security database storage structure with a data verification mechanism to complete data management. A Korean team implemented the blockchain PHR management platform; however, the data transaction time in their study was too long. To allow for the management of queries by a large number of patients, transaction and propagation times must improve [19]. Ahmed et al [20] proposed a blockchain-based emergency access control management system that can protect PHRs using a smart-contract design; however, the system manager can still retrieve real patient data, making privacy issues a concern. The platform designed in this study could offer patient-centered clinical record exchange and decision-making support and allow patients to view and share their own PHRs, as well as manage their health status and apply for medical data using other functions effectively. The platform and architecture could enable the meaningful use of PHRs and promote self-health management. The feasibility was demonstrated by an application test with international users in this study.

An important element of precision medicine is the exchange and management of PHRs and the subsequent provision of personalized medical treatment based on that data during the clinical diagnosis and treatment. This study therefore combined blockchain architecture and data verification methods to effectively solve the problems of data security, storage, and transmission and proposed a hybrid blockchain and data security approach that could enable effective international PHR exchanges. Using the AeHIN’s cross-national network environment, PHRs were successfully exchanged, and an international network of medical and health care providers was established to improve the quality of health care and precision medicine internationally.

### Principal Findings

The principal findings are as follows:

- A cross-country platform for PHRs was developed in this study. By using this platform, PHRs could be exchanged and shared between different organizations and individuals (doctors, patients, etc) in an efficient manner.
- A PHR platform was built using a blockchain architecture to ensure the security and privacy of health data. Few PHR systems based on blockchain technology have been developed for cross-country data exchange purposes.
- The platform has been tested by several users in different countries in the AeHIN and has shown that it is a suitable platform for PHR sharing and exchange.
- In our design, health data that can be used for precision medicine and can be stored and modeled in the architecture.

### Limitations

Currently, our PHR platform is at the prototype stage. Users from limited groups are participating in testing of the platform. However, the hardware architecture will need to be expanded to ensure the good performance of the platform when a large number of users wish to access the system. Furthermore, as the contents of the PHRs will be exchanged and shared by different countries and regions, an international data standard, such as HL7 FHIR, will be required to ensure smooth implementation.

### Future Directions

Important points regarding the comparison with prior work are as follows:

- Precision medicine is the future trend of health care and must be based on PHRs. Our PHR platform not only enables PHRs to be shared between countries but also creates space for future functions of precision medicine.
- Blockchain technology ensures data security and privacy and has been successfully used in financial data management systems.
- A cross-country medical care architecture must be developed in the present busy international activities.

### Conclusions

On the basis of the blockchain technology, it is possible to remove all limitations to patients’ ability to copy and transfer their own health records to other health service providers [21]. After data are uploaded in the blockchain, the block can guarantee that the records cannot be modified by anyone [22]. The PHRs are stored in a decentralized network; therefore, it is impossible to steal PHR data or hack the system illegally [21]. In addition to improved health record sharing and analysis, data sharing will be secured and privacy will be protected [23].

In addition, the blockchain technology is essential for future precision medicine applications. Through the blockchain architecture, the data required by precision medicine can be integrated from different sources. In addition to using blockchains as a ledger for patient care data, they can also be used to store various types of health care–related data, such as precision medical data and genomic data [24], health care plan data, patient-centered data [25], clinical trial data [26], medication supply chain data, and biomarker data [27-29]. In this study, we implemented a cross-country platform for PHRs. By using this platform, PHRs can be exchanged and shared between different organizations in an efficient manner. The platform has been tested by several users in different countries in the AeHIN and has been shown to be a suitable platform for PHR sharing and exchange. With our design, the health data that can be used for precision medicine can be stored and further modeled in the architecture. The security and privacy of PHRs can also be ensured by the features of blockchain technology, such as distributed node consensus algorithms, data transmission cryptography, and a decentralized network of smart contracts. However, an international standard, such as FHIR, will be required to ensure the PHR contents are internationally compatible.

## Abbreviations

AeHIN: Asia eHealth Information Network
ECC: elliptic curve cryptography
FHIR: Fast Healthcare Interoperability Resource
EMR: electronic medical record
MHB: My Health Bank
NHI: National Health Insurance
PAU: private accessible units
PHR: personal health record
PoA: proof of authority
RSA: Rivest-Shamir-Adleman
